# Sex Differences in Bipolar Disorders: Impact on Psychopathological Features and Treatment Response

**DOI:** 10.3389/fpsyt.2022.926594

**Published:** 2022-06-10

**Authors:** Giulia Menculini, Luca Steardo, Tiziana Sciarma, Martina D'Angelo, Laura Lanza, Gianmarco Cinesi, Federica Cirimbilli, Patrizia Moretti, Norma Verdolini, Pasquale De Fazio, Alfonso Tortorella

**Affiliations:** ^1^Section of Psychiatry, Department of Medicine and Surgery, University of Perugia, Perugia, Italy; ^2^Psychiatric Unit, Department of Health Sciences, University Magna Graecia of Catanzaro, Catanzaro, Italy; ^3^Local Health Unit Umbria 1, Department of Mental Health, Mental Health Center of Perugia, Perugia, Italy

**Keywords:** bipolar disorders, sex differences, psychopathology, treatment response, mood stabilizers

## Abstract

**Introduction:**

Sex differences were demonstrated in bipolar disorders (BD) concerning epidemiological, clinical, and psychopathological characteristics, but consensus is lacking. Moreover, data concerning the influence of sex on treatment response in BD is contrasting. The present cross-sectional study aimed to analyze sex differences in a population of BD subjects, with specific focus on psychopathological features and treatment response.

**Materials and Methods:**

Subjects diagnosed with BD according to the Diagnostic and Statistical Manual of Mental Disorders, 5th version (DSM-5) were recruited. Socio-demographic and clinical characteristics were collected. The Hamilton Rating Scale for Depression, the Mania Rating Scale (MRS), the brief version of the Temperament Evaluation of Memphis, Pisa and San Diego—Münster version (briefTEMPS-M), and the Barratt Impulsiveness Scale−11 items (BIS-11) were used for psychopathological assessment. Treatment response was appraised with the Alda Scale. We performed bivariate analyses to compare socio-demographic, clinical, and psychopathological characteristics between men and women (*p* < 0.05). A logistic regression was run to analyze features that were significantly associated with female sex.

**Results:**

Among the recruited 219 BD subjects, 119 (54.3%) were females. Women had a lower scholarity (*p* = 0.015) and were less frequently employed (*p* = 0.001). As for psychopathological features, a higher MRS total score (*p* < 0.001) was detected among women, as well as higher BIS-11 total score (*p* = 0.040), and briefTEMPS-M score for anxious temperament (*p* = 0.006). Men showed higher prevalence of DSM-5 mixed features (*p* = 0.025), particularly during a depressive episode (*p* = 0.014). Women reported longer duration of untreated illness (DUI) (*p* < 0.001). There were no sex differences in the Alda Scale total score when considering the whole sample, but this was significantly higher among men (*p* = 0.030) when evaluating subjects treated with anticonvulsants. At the logistic regression, female sex was positively associated with longer DUI (*p* < 0.001; OR 1.106, 95% CI 1.050–1.165) and higher MRS total score (*p* < 0.001; OR 1.085, 95% CI 1.044–1.128) and negatively associated with employment (*p* = 0.003; OR 0.359, 95% CI 0.185–0.698) and DSM-5 mixed features (*p* = 0.006; OR 0.391, 95% CI 0.200–0.762).

**Conclusions:**

The clinical presentation of BD may differ depending on sex. The severity of BD should not be neglected among women, who may also display worse treatment response to anticonvulsants.

## Introduction

Bipolar disorders (BD) are severe and potentially disabling psychiatric conditions with a prevalence of about 1.5% worldwide (1, 2). BD are characterized by manic, hypomanic, and depressive episodes, impairing mood, thinking, and psychomotor behavior (3, 4). These conditions more frequently present a chronic and relapsing course (5), with subsequent psychosocial functioning impairment and low quality of life (6–8).

The clinical phenomenology of BD is heterogeneous and depends on illness characteristics, such as BD type, but may also be influenced by several sociodemographic and environmental factors. Increasing interest has been dedicated to sex differences in BD clinical expression and course. Sex differences were already demonstrated for other categories of psychiatric disorders, particularly schizophrenia spectrum disorders, where they appeared to critically impact clinical features (9), outcomes (10, 11), and overall functioning (12). As for BD, previous research underlined differences in age at onset of the first manic episode, which was found to be lower in men (13), and in the prevalence of substance use disorder (SUD) comorbidity (14) and legal concerns (15), which was higher among men. On the other side, women showed a higher rate of rapid cycles (16, 17), mixed features (18, 19), and a higher number of depressive episodes (20) and suicide attempts (21, 22). Another study (23) identified that cognitive performances differed among women and men. Indeed, men performed better in working memory, whereas for women better results were evidenced in verbal learning and memory recognition tasks. As for treatment features, preliminary results showed that men were more often prescribed lithium (24), and women received antidepressant treatment in a higher percentage of cases (25). Despite this, studies focusing on sex differences in treatment features and treatment response in BD are scant. Preliminary results highlighted that women are more likely to respond to lithium (26), but the consensus is lacking. Indeed, previous research also showed that the effectiveness of maintenance therapies in BD was similar between the two sexes (27).

Concerning psychopathological features, there is a lack of studies assessing differences in affective temperaments and impulsivity, even if both are proved to be a predictive factors of higher psychopathological burden and poor treatment response in BD. Affective temperaments are trait-related precursors or subclinical manifestations of mood disorders. It was demonstrated that affective temperaments might influence specific psychopathological features, e.g., suicide risk (28), predominant polarity (29), mixed features (30), and overall functioning (31). Affective temperaments were also shown to influence response to lithium treatment (32). Sex differences in affective temperaments were highlighted in the general population, with a higher prevalence of anxious temperament among women (33), but research focusing on BD is scant (29). Previous reports did not detect significant differences in trait impulsivity when comparing men and women suffering from BD (34, 35), but the possible association between sex and impulsiveness deserves further attention. Indeed, impulsiveness is a highly prevalent clinical feature in BD, particularly during manic and mixed episodes (36). However, it can also be considered a core psychopathological dimension in this population of subjects, possibly associated with higher number of episodes and suicide attempts (34, 37). Since trait characteristics lie on a biological basis, their possible association with sex could help differentiate the psychopathological picture of BD even since illness onset, thus guiding possible prevention and treatment strategies.

The primary aim of the present study was to evaluate sex differences in a population of subjects suffering from BD, focusing on clinical and psychopathological characteristics. As for the latter, special attention was paid to trait-like characteristics, particularly impulsivity and affective temperaments. We hypothesized that sex could affect the prevalence and expression of such characteristics. The secondary aim was to compare treatment response to mood stabilizers among men and women suffering from BD. To our knowledge, previous research on the topic did not use standardized assessment tools for evaluating response to treatment. Further insights into sex-based differences in treatment response would be of critical aid in implementing personalized treatment approaches for BD subjects.

## Materials and Methods

### Study Participants and Procedures

This cross-sectional study was conducted in a naturalistic setting between April 1st, 2018, and December 31st, 2021. Subjects were consecutively recruited at the outpatient service of the Psychiatric Unit of the University “Magna Graecia” of Catanzaro, Italy, and the outpatient and inpatient services of the Psychiatric Unit of the General Hospital/University of Perugia.

Subjects aged ≥18 years with a diagnosis of BD according to the Diagnostic and Statistical Manual of Mental Disorders, 5th edition (DSM-5) (38) were consecutively invited to participate in the study. Subjects were excluded in case of moderate/severe cognitive impairment, comorbidity with medical diseases that could affect the psychopathological condition, and insufficient comprehension of written/oral Italian language.

The diagnoses were carried out using the Structured Interview for DSM-5 Disorders, Clinician Version (SCID-5-CV) (39), administered by trained psychiatrists.

Subjects who met the inclusion criteria were given a complete explanation of the study protocol and provided written informed consent for participation in the study. Subsequently, they underwent a study visit during which we collected information concerning the study variables and administered psychometric assessment tools. An *ad hoc* schedule was used to collect: socio-demographic (biological sex, age, education, working status, living status), clinical (psychiatric diagnosis, psychiatric comorbidities, familiarity for psychiatric disorders, age at onset of the first affective episode, number of episodes, presence/absence of a predominant polarity—defined as at least two thirds of the previous mood episodes belonging to the same polarity (40), number of hospitalizations, seasonality, lifetime suicide attempts, psychotic symptoms, aggressiveness, anxiety features, current mixed features according to the DSM-5 specifier, treatment features, antidepressant-induced mania, and response to treatment with mood stabilizers), and psychopathological (severity of depressive and hypomanic/manic symptoms, predominant affective temperament, and impulsiveness) characteristics.

During the study visit, the severity of depressive and hypomanic/manic symptoms was appraised by the Hamilton Rating Scale for Depression (HRSD) (41, 42) and the Young Mania Rating Scale (YMRS) (43, 44). The HRSD is a 21-item, clinician-administered tool evaluating different dimensions of depressive symptoms, both somatic and non-somatic, during the 15 days prior to evaluation. The total score is obtained by the sum of the first 17 items, while the remaining 4 are used for characterizing specific subtypes of depression. Higher scores underpin higher depression severity. The YMRS is composed of 11 items and assesses core symptoms of mania during the previous 48 h. Items are scored following a 0–4-point scale, except for irritability, speech, thought content, and disruptive/aggressive behavior, graded on a 0–8 scale. The sum of all items represents the total score, and higher scores correspond to higher symptom severity.

Predominant affective temperaments were evaluated with the brief Temperament Evaluation of Memphis, Pisa and San Diego—Münster version (briefTEMPS-M) (45, 46). The briefTEMPS-M is an auto-administered scale composed of 35 items, which identify the five affective temperaments (depressive, cyclothymic, hyperthymic, irritable, and anxious). The total score for each temperament is calculated by adding up the items. The tool was validated in the Italian language, and mean scores are available for both clinical and non-clinical populations (46, 47).

Lifetime impulsiveness was evaluated with the Italian version of the Barratt Impulsiveness Scale, 11 items (BIS-11) (48, 49), a self-report tool of 30 items that explore different dimensions of impulsiveness, namely attentional, motor, and non-planning impulsiveness. Items are scored on a 4-point Likert scale and higher scores indicate the severity of impulsiveness.

Response to mood stabilizer treatment was assessed using the Alda Scale, also known as retrospective criteria of long-term treatment response in BD. The scale consists of two clinician-evaluated criteria: (A) rating of the association between clinical improvement and mood stabilizer treatment, and (B) rating of the strength of the association between clinical improvement and mood stabilizer treatment. The total score can be obtained by subtracting the B score from the A score and varies from 0 to 10. A total score range of 10–7 is considered as a good response to mood stabilizers, 6–4. As a moderate response, and 3–0 as a lack of response. The Alda Scale total score was considered in the present study both as a continuous and a dichotomous variable. For the latter, a total score ≥7 was considered as indicative of a good response to mood stabilizers, while a total score <7 was considered for lack of good treatment response (50, 51).

The study was carried out in accordance with the Declaration of Helsinki principles and was approved by the Ethics Committee of the Umbria and Calabria Regions (protocol N. Umbria 12958/18/ON; Calabria 307).

### Statistical Analysis

The collected data was entered into an electronic dataset created by the Psychiatric Unit of the University of Catanzaro. We carried out descriptive analyses to evaluate the distributional properties of the variables in the samples. Categorical variables were reported as frequencies and absolute percentages, while continuous variables were expressed as mean ± standard deviation (SD) or median and interquartile range (IQR). The normality of the distribution was assessed by using the Kolmogorov-Smirnov test. Subsequently, subjects were divided into two groups according to sex. After verifying the assumptions for running the tests, the two groups were compared by means of the Chi-square test for categorical variables and the Student's *t*-test for continuous variables.

Response to treatment was also assessed in different subgroups created according to the type of mood stabilizer treatment, namely lithium or anticonvulsants (valproate/carbamazepine). We did not perform the sub-analysis for lamotrigine and other mood stabilizers (e.g., atypical antipsychotics with mood stabilizing properties) due to the small sizes of these sub-samples. We chose a parametric technique for bivariate analyses involving continuous variables due to its sensitivity and property of guaranteeing sufficient robustness even when the normality assumptions are violated, if the sample size is adequate(>30 for each subsample) (52–54). For the subsample analyses concerning different drugs, we performed the Mann-Whitney U test if the distribution of the continuous variable violated the assumption of normality. All tests were two-tailed, and the significance level was set as p <0.05. We chose not to apply a *p*-value correction for multiple comparisons (e.g., Bonferroni correction) to avoid type II errors. Indeed, the main aim of the study was to test an hypothesis mainly concerning two groups of variables, namely psychopathological and treatment features, and our purpose was not to miss possible significant associations that could be worthy of further exploration (55).

A logistic regression model was run to evaluate significant associations between female sex and the independent variables that showed significance at the bivariate analyses. All tolerance values in the regression analyses were >0.1 and all variance inflation factors were <10, expressing that the assumption of multicollinearity was not violated. Odds ratios (OR) with 95% confidence intervals were assessed for observed associations. Statistical analyses were performed using the Statistical Package for Social Sciences (Statistical Package for Social Science-SPSS, 26.0 version for Windows Inc., Chicago, IL, USA).

## Results

### Description of Sample Characteristics

In the present study, we included 219 subjects suffering from BD, among which 119 (54.3%) were females. The median age was 48 years old (IQR 21). The majority of subjects in the sample were single (*n* = 108, 49.3%), had a scholarity of at least 13 years (*n* = 166, 65.8%), and were employed (*n* = 120, 54.8%). Most subjects were outpatients (*n* = 168, 76.7%) and the main diagnosis was type I BD (*n* = 141, 64.4%).

As for treatment features, 179 (82.6%) subjects were prescribed mood stabilizer monotherapy with lithium or anticonvulsants, while 7 (3.2%) were taking more than one mood stabilizer. Particularly, 98 (44.7%) subjects were on lithium, 76 (34.7%) were prescribed valproate or carbamazepine (*n* = 66 valproate, *n* = 10 carbamazepine), and 7 (3.2%) assumed lamotrigine. Furthermore, 31 (14.2%) subjects were prescribed atypical antipsychotics with mood stabilizing properties (e.g., quetiapine).

### Sex Differences in Sociodemographic, Clinical, and Psychopathological Characteristics

No differences in age and marital status were found comparing sociodemographic characteristics in the two subgroups of BD subjects (men vs. women). Men had a higher scholarity since they more frequently completed high school (84 vs. 68.9%, *p* = 0.015) and were employed in a higher percentage of cases (68 vs. 43.7%, *p* = 0.001). No significant differences were detected when comparing diagnostic features, including psychiatric comorbidity.

As for clinical characteristics, no differences in the current affective episode were detected. Men showed a significantly higher prevalence of current DSM-5 mixed features (59 vs. 42.9%, *p* = 0.025). Particularly, depression with mixed features was significantly more frequent among men (66.7 vs. 40%, *p* = 0.014). When evaluating symptom severity with the HRSD and MRS, the severity of mania, but not the severity of depression, was significantly higher in women (MRS mean total score 19.98 ± 10.20 vs. 14.43 ± 9.65, *p* < 0.001). The BIS-11 mean total score was significantly higher women as well (77.12 ± 12.6 vs. 71.16 ± 17.52, *p* = 0.006), with no significant differences between the three BIS-11 subscales (attentional, motor, and non-planning impulsiveness). As for affective temperaments, the briefTEMPS-M mean score for anxious temperament was significantly higher in women (19.74 ± 9.12 vs. 17.50 ± 6.02, *p* = 0.040). The DUI was significantly longer among women (mean 7.62 ± SD 10.82 vs. 2.85 ± 5.56, *p* < 0.001). For between-sex comparisons see Table 1.

**Table 1:** Comparison of socio-demographic, clinical, and psychopathological characteristics between females (*n* = 119, 54.3%) and males (*n* = 100, 45.7%) in our sample.

**Socio-demographic and clinical characteristics**					
	**Females (** ***n*** **, %)**	**Males (** ***n*** **, %)**	**χ^2^**	***p***	**OR (CI 95%)**
Marital status: single	56 (47.9)	52 (57.8)	1.626	0.202	1.491 (0.857–2.593)
Scholarity ≥13 years	82 (68.9)	84 (84)	5.950	***0.015***	2.369 (1.224–4.586)
Employed	52 (43.7)	68 (68)	11.994	***0.001***	2.738 (1.572–4.768)
BD-I (yes listed)	75 (63)	66 (66)	0.100	0.752	1.139 (0.653–1.987)
Current depressive episode	55 (46.2)	45 (45)	0.000	1.000	0.970 (0.568–1.656)
Current manic/hypomanic episode	52 (43.7)	43 (43)	0.000	1.000	0.989 (0.578–1.694)
Familiar psychiatric history	71 (59.7)	59 (59)	0.000	1.000	0.997 (0.579–1.717)
Psychiatric comorbidity	55 (46.6)	45 (45)	0.010	0.919	0.937 (0.549–1.600)
Seasonality	60 (52.2)	37 (37)	3.627	0.057	0.565 (0.326–0.979)
History of predominant polarity	41 (36.3)	29 (29)	0.530	0.467	0.772 (0.432–1.380)
Antidepressant–induced mood switch	32 (28.8)	25 (25)	0.003	0.955	0.935 (0.505–1.733)
History of suicide attempts	39 (32.8)	30 (30)	0.086	0.769	0.879 (0.495–1.561)
Anxiety symptoms	55 (46.2)	51 (51)	0.324	0.569	1.211 (0.711–2.063)
Psychotic symptoms	48 (40.7)	49 (49)	1.200	0.273	1.401 (0.819–2.398)
Aggressiveness	65 (54.6)	51 (51)	0.059	0.808	0.901 (0.527–1.541)
DSM-5 mixed features	51 (42.9)	59 (59)	5.037	***0.025***	1.919 (1.119–3.290)
SUD	45 (37.8)	36 (36)	0.019	0.891	0.925 (0.533–1.605)
	**Females (mean, SD)**	**Males (mean, SD)**	***t***		***p***
Age	47.44 (12.93)	45.23 (14.87)	1.175		0.241
Age at onset	25.68 (9.08)	26.78 (12.04)	−0.769		0.442
Number of episodes	13.11 (16.25)	11.41 (13.41)	0.822		0.412
Number of hospitalizations	2.47 (9.85)	1.77 (8.77)	0.551		0.582
DUI (years)	7.62 (10.82)	2.85 (5.56)	3.982		*** <0.001***
**Psychopathological features**					
MRS total score	19.98 (10.20)	14.43 (9.66)	4.111		*** <0.001***
HRSD total score	21.69 (11.81)	20.73 (13.54)	0.044		0.960
BIS-11 total score	77.12 (12.60)	71.16 (17.51)	2.827		***0.006***
BriefTEMPS-M depressive	22.87 (9.39)	21.17 (7.09)	1.456		0.147
BriefTEMPS-M cyclothymic	22.91 (9.76)	20.66 (7.79)	1.815		0.071
BriefTEMPS-M hyperthymic	20.42 (8.97)	20.39 (7.76)	0.031		0.975
BriefTEMPS-M irritable	19.22 (9.77)	19.33 (8.04)	−0.985		0.932
BriefTEMPS-M anxious	19.74 (9.10)	17.50 (6.03)	2.066		***0.040***
Alda scale total score	6.03 (13.51)	4.40 (2.71)	1.189		0.236

*BD-I, Bipolar Disorder type 1; BIS-11, Barratt Impulsiveness Scale-11 items; BriefTEMPS-M, Brief Temperament Evaluation of Memphis, Pisa, San Diego; DSM-5, Diagnostic and Statistical Manual of Mental Disorders, 5th Edition; DUI, Duration of Untreated Illness; HRSD, Hamilton Rating Scale for Depression; MRS, Mania Rating Scale; SUD, Substance use disorder*.

*For all categorical variables, “yes” are listed*.

*Significant p-values (<0.05) are reported in bold and italics*.

### Sex Differences in Treatment Features and Response to Treatment With Mood Stabilizers

Men were more frequently prescribed lithium when assessing differences in treatment features (64 vs. 42.2%, *p* = 0.005), while no significant differences were found for anticonvulsant monotherapy. As for the response to treatment with mood stabilizers, the Alda Scale mean total score did not change among men and women when considering the whole sample. The response to lithium treatment was not significantly different when dividing the sample based on the prescribed mood stabilizer therapy. On the contrary, in the subpopulation of subjects treated with anticonvulsants, the total score at the Alda scale was significantly higher among men (median 5, IQR 6 vs. median 3, IQR 5, *p* = 0.030), underpinning a better treatment response. When the Alda scale was used as a dichotomous variable, response to treatment did not significantly differ among women and men.

### Sociodemographic and Clinical Variables Significantly Associated With Female Sex

At the logistic regression, female sex was set as the dependent variable and scholarity > 8 years, working status (employment), DUI, DSM-5 mixed features, MRS and BIS-11 total scores, and briefTEMPS-M anxious temperament score were entered as independent variables. The model (χ^2^ = 63.094, df = 7, *p* < 0.001) explained between 26.5% (Cox and Snell's R square) and 35.3% (Nagelkerke R square) of the variance. Female sex was significantly associated with longer DUI (*p* < 0.001, OR 1.106, 95% CI 1.050–1.165) and higher MRS total score (*p* < 0.001, OR 1.085, 95% CI 1.044–1.128), which displayed a positive association, as well as with employment (*p* = 0.003, OR 0.359, 95% CI 0.185–0.698) and DSM-5 mixed features (*p* = 0.006, OR 0.391, 95% CI 0.200–0.762), which demonstrated a negative association (see Table 2).

**Table 2:** Logistic regression model evaluating socio-demographic, clinical, and psychopathological variables associated with female sex in our sample.

**Variables in equation**	**Wald**	***p*-value**	**OR (95% CI)**
BIS-11 total score	0.263	0.608	0.993 (0.968–1.019)
BriefTEMPS-M anxious	2.169	0.141	1.025 (0.989–1.084)
DSM-5 mixed features	7.591	***0.006***	0.391 (0.200–0.762)
DUI	14.274	*** <0.001***	1.106 (1.050–1.165)
MRS total score	16.942	*** <0.001***	1.085 (1.044–1.128)
Scholarity ≥13 years	0.000	0.996	1.002 (0.444–2.261)
Working status: employed	9.134	***0.003***	0.359 (0.185–0.698)

*Chi-square: 63.094; df: 7; p < 0.001*.

*BIS-11, Barratt Impulsiveness Scale-11 items; BriefTEMPS-M, Brief Temperament Evaluation of Memphis, Pisa, San Diego; DSM-5, Diagnostic and Statistical Manual of Mental Disorders, 5th Edition; DUI, Duration of Untreated Illness; MRS, Mania Rating Scale. Significant p-values for the observed associations are reported in bold and italics*.

## Discussion

The present BD sample was composed of a slightly higher number of women, similarly to what reported by previous research (22). Despite BD has traditionally been described as a psychiatric disorder with no sex differences in terms of lifetime prevalence (56), recent evidence demonstrated a predominance of female sex among several studies based on large BD populations (57). The overall educational level in our sample was high, confirming previous literature that showed a similar scholarity between BD subjects and the general population (58). Despite this, lower education and higher unemployment rates were detected among women in our population, the latter being significantly associated with female sex at the logistic regression. Our results are consistent with previous research (59, 60) and may be considered as indicators of worse psychosocial outcomes and lower functioning—since occupational status is listed among the Functioning Assessment Short Test domains (61)—in women suffering from BD (62).

When investigating sex differences in clinical and psychopathological characteristics, women displayed a higher severity of mania, while men more often presented an affective episode with mixed features. As for trait-like psychopathological characteristics, women showed higher impulsiveness and predominant anxious affective temperament, but these associations were not significant at the logistic regression. The finding on the severity of mania is partially consistent with previous literature. Indeed, although depressive episodes were found to be more frequent among women suffering from BD (63), it has also been reported that they were more often hospitalized for mania (64, 65), suggesting higher severity of manic symptoms. These results should be taken into account by clinicians also in consideration of the fact that manic symptoms were demonstrated to predict BD relapses during pregnancy and the post-partum period (66) and thus deserve special attention in women suffering from BD.

Our result regarding mixed features was partially unexpected since, in previous research, mixed episodes were more frequently reported among men, despite the lack of univocal results (67). Contrasting findings could be explained by the different tools used for assessing mixed features (e.g., following the DSM-IV-TR or ICD-10 criteria for mixed episodes rather than the DSM-5 mixed features specifier) (67). Moreover, evidence concerning mixed features in women suffering from BD was mainly reported for mixed mania (19, 68, 69), while in our study the difference mainly concerned mixed depression. This result would suggest to carefully assess the presence of mixed symptoms when evaluating men suffering from BD during depressive phases. Indeed, the presence of mixed features in depression is associated with higher suicide risk (70), aggressiveness (71), as well as with a higher risk of substance use (72, 73). Subsequently, the early recognition of mixed states during depressive episodes is crucial for addressing adequate treatment strategies (74, 75). The finding concerning trait-like impulsivity may be consistent with the higher prevalence of personality disorders (22), particularly cluster B disorders (76), among women who suffer from BD. It should also be underlined that women in the present sample displayed a higher severity of manic symptoms, which could partially explain findings concerning impulsiveness. This result deserves to be further explored, also in consideration of the relevant association between impulsivity and suicidality in BD (77). Similarly, the higher liability to a predominant anxious affective temperament should be further investigated. Indeed, sex differences in predominant affective temperament are still poorly understood and findings from previous research are contrasting (29). This issue could be due to the partial overlap of psychopathological features associated with different affective temperaments, which is supported by the evidence of a “depressive-cyclothymic-anxious-irritable” affective temperamental disposition in BD, underpinning higher emotional liability and suicide risk (28, 29, 78). Anxious temperament is linked to a higher prevalence of anxiety disorders among women (79), consistent with the more frequent comorbidity between anxiety disorders and BD in the female sex (80). Despite this, the correlates of anxious temperament in BD still need to be clarified, and previous literature suggested that this predominant temperament could affect the clinical picture of BD without representing a predictor of poorer outcomes (81).

In the present sample, both males and females revealed a mean long DUI, defined by previous reports as >2 years (82), and a significant positive association between DUI and female sex was revealed by the logistic regression. A longer DUI underpins worse BD outcomes, as demonstrated by a higher number of mood episodes, suicide attempts, and hospitalizations (83, 84) and a higher prevalence of psychiatric and medical comorbidities (85, 86). Moreover, delayed treatment in affective disorders is associated with a worse response to pharmacological and psychological interventions (87–89). For this reason, early engagement with mental health services might be crucial for reducing DUI, as already demonstrated for psychotic disorders (90). The longer DUI could explain, at least in part, our findings concerning worse response to anticonvulsant treatment in women. To our best knowledge, this is one of the first studies assessing response to mood stabilizer treatment in subjects suffering from BD by using a validated tool. Previous studies investigated differences in treatment response as evaluated by indirect indexes, e.g., rehospitalizations (27). The lack of significant sex differences in response to mood stabilizers already emerged from prior research (27). When stratifying subjects according to the prescribed drug, better treatment response to anticonvulsants was demonstrated among men, despite the Alda scale score were classified in an intermediate response range for both sexes. This finding deserves further clarification and should be taken into account by clinicians. Indeed, valproate treatment is associated with teratogenic risk and with menstrual irregularities (91) and may also present pharmacodynamic interactions with hormonal contraceptives (92). The possible lower response to valproate treatment among women, together with the iatrogenic morbidity associated with this treatment, could thus suggest its prescription only in selected cases. Further in-depth knowledge on sex differences in response to treatment is also needed since treatment of BD in female subjects presents major challenges, e.g., due to hormonal variations during pregnancy and post-partum.

In the present study we did not highlight any significant sex differences in suicidality, comorbid substance use disorders, rapid cycling, polarity, and age at onset. This finding is partly contrasting with previous literature, which could be explained by high among-studies variability in settings and sample characteristics. Moreover, some reports did not show any difference among the above mentioned characteristics (22, 59). Previous research underlined the absence of significant associations between sex and markers of poor clinical outcome in BD (60), suggesting that future studies should further explore such differences in BD.

The results of the present study should be evaluated taking into account its limitations. First of all, the sample size is relatively small and may limit the generalizability of the findings. Unfortunately, the number of subjects who were treated with lamotrigine or atypical antipsychotics did not allow performing bivariate analysis for assessing sex differences in response to treatment with these drugs. At the logistic regression, OR for significant variables were close to 1, suggesting that the observed associations should be further investigated by future research. As for the study setting, subjects were recruited at two University Hospitals, so the population is not fully representative of BD subjects in our catchment areas. Moreover, it should be underlined that we only collected data on biological sex and did not take into account gender when analyzing the results.

Overall, despite the discussed limitations, results from the present study may help mental health professionals in the personalized clinical management of BD. Special attention should be paid to the presence of a possible bipolar diathesis in women at their first depressive episode, given the association with a longer DUI, and to the presence of adequate pathways to care for this population. Moreover, integrated interventions oriented toward better psychosocial outcomes should always be evaluated for women with BD, while the choice of mood stabilizer treatment should consider the higher severity of manic symptoms in this population.

## Conclusions

Specific sociodemographic, psychopathological, and course features significantly differed between women and men in our sample, suggesting that sex may affect the overall clinical picture of BD. Remarkably, higher severity of manic symptoms and worse response to anticonvulsants advise that the severity of bipolar illness should not be neglected among women. Poorer socio-economic outcomes and the longer DUI in women also suggest that equality in access to care should remain a priority in the treatment of serious mental illnesses.

## Data Availability Statement

The raw data supporting the conclusions of this article will be made available by the authors, without undue reservation.

## Ethics Statement

The studies involving human participants were reviewed and approved by Comitato Etico Regione Umbria and Comitato Etico Regione Calabria. The patients/participants provided their written informed consent to participate in this study.

## Author Contributions

GM, LS, and TS conceived the idea and designed the study. MD'A, LL, GC, and FC collected the data. GM performed the statistical analysis. GM, LS, LL, and GC wrote the original draft. TS, MD'A, FC, NV, PM, PD, and AT revised the whole manuscript. AT supervised the study during all its phases. All authors contributed to the article and approved the submitted version.

## Conflict of Interest

GM has received travel grants from Janssen unrelated to the present work. NV has received financial support for CME activities and travel funds from Angelini, Janssen-Cilag, Lundbeck, Otsuka unrelated to the present work. AT has received research support from Lundbeck and served as speaker for Lundbeck and Angelini unrelated to the present work. The remaining authors declare that the research was conducted in the absence of any commercial or financial relationships that could be construed as a potential conflict of interest.

## Publisher's Note

All claims expressed in this article are solely those of the authors and do not necessarily represent those of their affiliated organizations, or those of the publisher, the editors and the reviewers. Any product that may be evaluated in this article, or claim that may be made by its manufacturer, is not guaranteed or endorsed by the publisher.
